# Characterization of six CaMKIIα variants found in patients with schizophrenia

**DOI:** 10.1016/j.isci.2021.103184

**Published:** 2021-09-27

**Authors:** Carolyn Nicole Brown, Sarah G. Cook, Hillary F. Allen, Kevin C. Crosby, Tarjinder Singh, Steven J. Coultrap, K. Ulrich Bayer

**Affiliations:** 1 Department of Pharmacology, University of Colorado Anschutz Medical Campus, Aurora, CO 80045, USA; 2 Program in Neuroscience, University of Colorado Anschutz Medical Campus, Aurora, CO 80045, USA; 3 Stanley Center for Psychiatric Genetics, Broad Institute of MIT and Harvard, Cambridge, MA 02142, USA

**Keywords:** Biological sciences, Neuroscience, Protein structure aspects

## Abstract

The Ca^2+^/Calmodulin-dependent protein kinase II (CaMKII) is a central regulator of synaptic plasticity and has been implicated in various neurological conditions, including schizophrenia. Here, we characterize six different CaMKIIα variants found in patients with schizophrenia. Only R396stop disrupted the 12-meric holoenzyme structure, GluN2B binding, and synaptic localization. Additionally, R396stop impaired T286 autophosphorylation that generates Ca^2+^-independent “autonomous” kinase activity. This impairment in T286 autophosphorylation was shared by the R8H mutation, the only mutation that additionally reduced stimulated kinase activity. None of the mutations affected the levels of CaMKII expression in HEK293 cells. Thus, impaired CaMKII function was detected only for R396stop and R8H. However, two of the other mutations have been later identified also in the general population, and not all mutations found in patients with schizophrenia would be expected to cause disease. Nonetheless, for the R396stop mutation, the severity of the biochemical effects found here would predict a neurological phenotype.

## Highlights

• Two of six CaMKII variants found in patients with schizophrenia showed impairments
• R396stop disrupted holoenzyme structure, T286 autophosphorylation, and GluN2B binding
• R8H reduced T286 autophosphorylation and stimulated activity
• Two of the four other variants were later found also in the general population

## Graphical abstract

**Figure:**
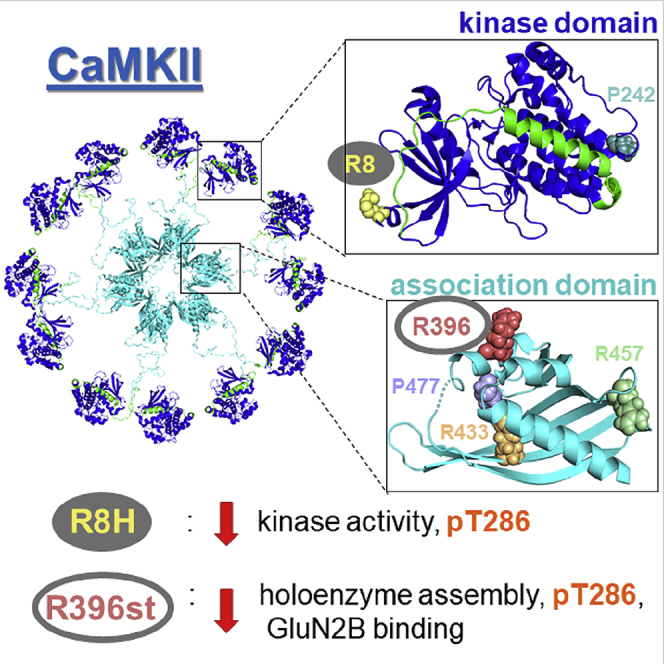

## Introduction

Schizophrenia is a chronic, debilitating, neuropsychiatric disorder that affects an individual's ability to interpret reality and function normally. Symptoms of schizophrenia are classified as negative (anhedonia), cognitive (impaired working memory), and positive (hallucinations; delusions) (McCutcheon et al., 2020; Owen et al., 2016). Positive symptoms are effectively targeted by most currently available antipsychotics, whereas negative and cognitive symptoms show only mild response even to second- and third-generation antipsychotics, despite their wide range of targets (Bilder et al., 2002; Goff et al., 2011; Kahn et al., 2015; Koster et al., 2014; Lee et al., 2013; Maroney, 2020). Thus, there is a major unmet need for antipsychotics that improve symptoms like cognitive dysfunction. Cognitive function such as learning and memory are thought to require long-term potentiation (LTP) of hippocampal synapses, which in turn requires the Ca^2+^/Calmodulin (CaM)-dependent kinase II (CaMKII). CaMKII dysregulation has been implied in several neurological and neurodegenerative diseases (Chia et al., 2018; Cook et al., 2019; Liu and Murray, 2012; Picconi et al., 2004) and has recently gained attention in neuropsychiatric disorders like schizophrenia (Frankland et al., 2008; Robison, 2014). For example, recent genetic studies have found schizophrenia-associated mutations in proteins common to CaMKII signaling, including Ca^2+^ channels (Cross-Disorder Group of the Psychiatric Genomics, 2013) and postsynaptic density (PSD) proteins (Fromer et al., 2014; Kirov et al., 2012). Furthermore, *CaMKIIα*^*+/*−^ mice have been described to have a schizophrenia-related phenotype, including aggressive behavior, immature dentate gyrus (DG), and impaired working memory (Yamasaki et al., 2008). Though this discovery was made over a decade ago, little progress has been made in understanding the potential role of CaMKII dysregulation in schizophrenia pathophysiology.

**Figure 1:**
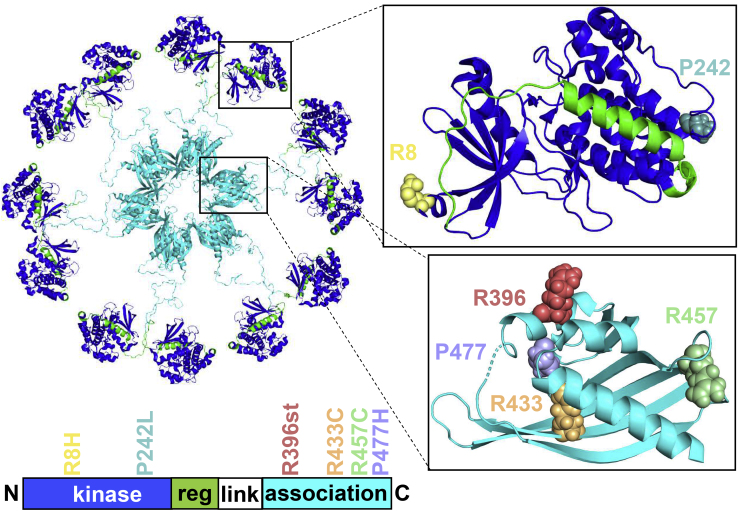
The CaMKII holoenzyme and mutations identified in patients with schizophrenia CaMKII 12-meric holoenzyme structure (PDB ID: 5u6y) with six mutations that were found in patients with schizophrenia by high-throughput exome sequencing. Two of the mutations (P242L and R457C) were later also detected in the healthy control population (see Table S1 and SCHEMA browser, https://schema.broadinstitute.org, gene name CAMK2A). Residues 7–12 from PBD ID: 3soa were superimposed on 5u6y. Residues 473–477 were built in PyMOL. Reg = regulatory, link = linker.

Here, we characterize six mutations that were found in the α-isoform of CaMKII in patients with schizophrenia by high-throughput exome sequencing (Figure 1, Table S1; see also https://schema.broadinstitute.org). Of these mutations, two are within the CaMKII kinase domain, which mediates the catalytic activity. The remaining four mutations are within the CaMKII association domain, which is required for formation of CaMKII 12-meric holoenzymes (for review see Bayer and Schulman, 2019; Bhattacharyya et al., 2020). Holoenzyme formation enables additional regulatory mechanisms that are essential for LTP (Cook et al., 2021; Coultrap et al., 2014a; Giese et al., 1998; Hanson et al., 1994). Briefly, in the basal state, the kinase domain is autoinhibited by the regulatory domain, which can be displaced by Ca^2+^/CaM. As a result, the substrate binding site is exposed, and the kinase becomes active. Additionally, T286 within the regulatory domain is exposed, enabling its phosphorylation by a neighboring subunit (“autophosphorylation”) within the holoenzyme (Hanson et al., 1994; Rich and Schulman, 1998). Displacement of the regulatory domain also exposes the T-site, enabling CaMKII to bind the GluN2B subunit of the NMDA receptor, and this binding also requires the CaMKII holoenzyme (Bayer et al., 2001; Strack et al., 2000). Both this binding to GluN2B and the T286 autophosphorylation keeps CaMKII in a partially activated “autonomous” state even after dissociation of Ca^2+^/CaM (Bayer et al., 2001; Miller and Kennedy, 1986). Importantly, both mechanisms are required for normal LTP (Giese et al., 1998; Halt et al., 2012). Interestingly, GluN2B mutations have also previously been associated with schizophrenia, including in the intracellular C-terminus that contains the CaMKII binding site (Myers et al., 2019).

We measured the effects of six CaMKII mutations found in patients with schizophrenia using a combination of spectroscopy, optogenetics, *in vitro* biochemistry, and live cell imaging. We demonstrate that the R8H kinase domain variant impairs stimulated CaMKII activity while the R396stop association domain mutation impairs holoenzyme formation, T286 autophosphorylation, GluN2B binding, and synaptic localization of CaMKII. Somewhat surprisingly, CaMKII expression levels were unaffected by any of the variants. These data highlight that single disease-associated mutations are very often not causal of the disease itself. Indeed, of the four mutations that did not have detectable effects on CaMKII, two were later found also in the general population. However, the severe biochemical effects of one of the mutations, R396stop, would be expected to result in a neurological phenotype.

## Results

### Association domain stop mutation ablates CaMKII holoenzyme formation

**Figure 2:**
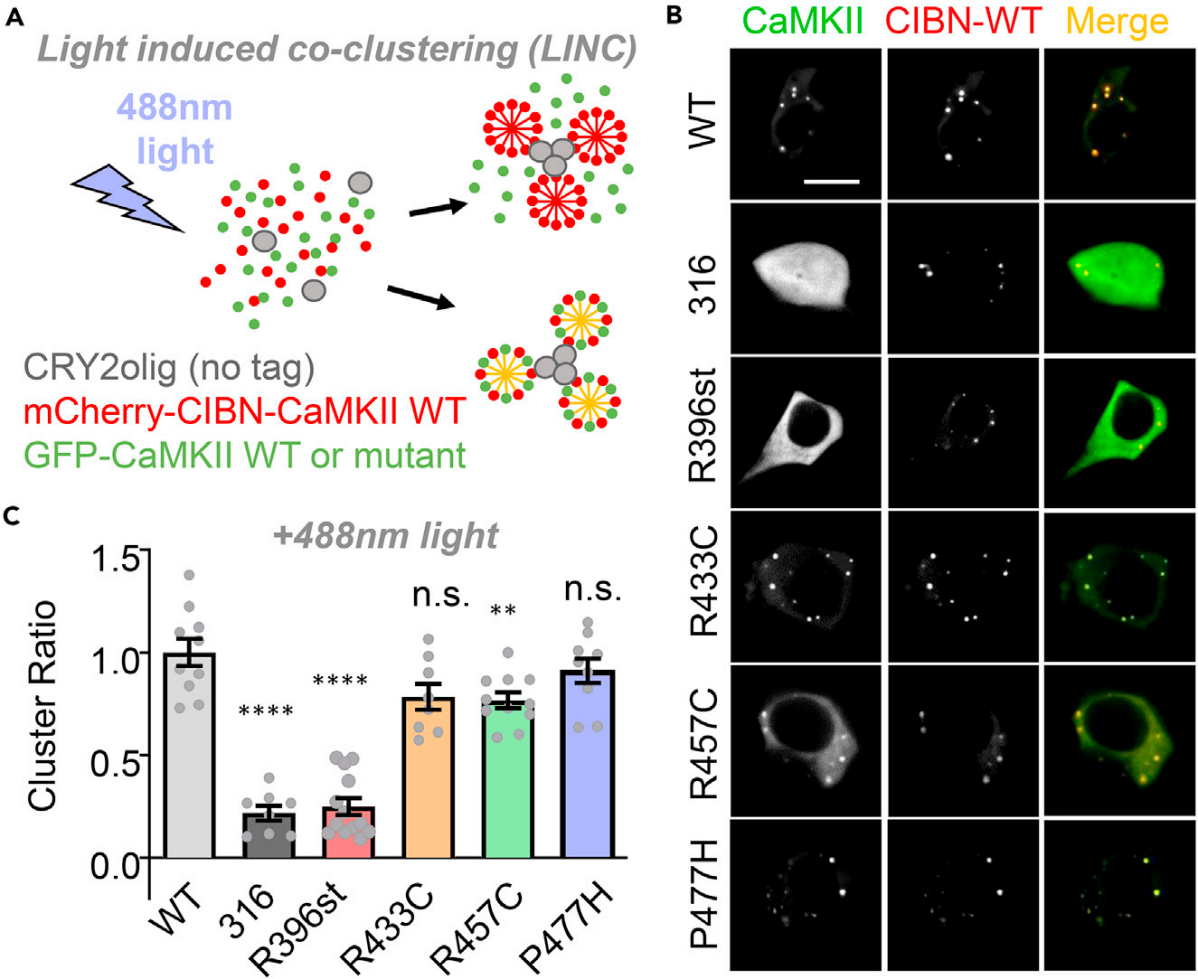
CaMKII stop mutation results in decreased subunit oligomerization (A) Optogenetic light-induced clustering (LINC) assay. CRY2olig, CIBN-mCherry(mCh)-CaMKII, and GFP-CaMKII are transfected into HEK293 cells. Stimulation with blue light causes both clustering of CRY2olig and recruitment of CIBN to CRY2olig. mCh^+^GFP^+^ (yellow) clusters indicate an interaction between CIBN-mCh-CaMKII and GFP-CaMKII. (B) LINC representative images (scale bar = 10μm). 316 is a CaMKII truncation mutant that completely lacks the association domain. (C) mCh/GFP co-localization quantitation; ∗∗p<0.01; ∗∗∗∗p<0.0001; n.s., not significant versus WT, in one-way ANOVA with Tukey's post hoc analysis. The dramatic effects of the monomeric 316 control and the R396st mutation were indistinguishable from each other, suggesting that both are monomers. Data are represented as mean ± SEM.

Six CaMKII mutations were identified in patients with schizophrenia and are found in the kinase and association domains (Figure 1; Table S1). The R396stop variant lacks a significant portion of the association domain and was thus expected to impair CaMKII holoenzyme formation. The remaining three association domain missense mutations do not occur in inter-subunit interacting residues but could indirectly impair holoenzyme formation through association domain unfolding. In patients, these spontaneous mutations were heterozygous. Thus, we first determined if the variants interact with wild-type (WT) CaMKII. To this end, a light-induced co-clustering (LINC) assay was used in human embryonic kidney (HEK) 293 cells (Figure 2A). This assay can be used to probe protein–protein interactions by the use of two light-activated clustering proteins, CRY2olig, and CIBN. The assay has been developed by Dr. Chandra Tucker's group and has been used previously by us and by others for detecting CaMKII holoenzyme formation (Cook et al., 2018; Taslimi et al., 2014). Untagged CRY2olig, CIBN-mCherry(mCh)-CaMKII WT, and different GFP-CaMKII variants are co-expressed in HEK293 cells. Stimulation with blue light induces clustering of CRY2olig and CIBN-mCh-CaMKII. If a co-expressed GFP-CaMKII can bind to the CIBN-mCh-CaMKII WT, it would co-cluster, as seen for GFP-CaMKII WT (Figures 2B and 2C; Video S1). Failure of GFP-CaMKII R396stop (R396st) to co-cluster, instead, indicates impaired interaction with WT CIBN-mCh-CaMKII (Figures 2B and 2C; Video S2). By contrast, the association domain missense mutations R433C, R457C, and P477H showed only minimal effects on co-clustering that were statistically significant only for the R457C mutation.

Video S1. Monitoring of light induced co-clustering of GFP-CaMKII WT with CIBN-mCh-CaMKII WT and untagged-Cry2olig expressed in HEK293 cells, related to Figure 2 Following blue-light stimulation, GFP-CaMKII WT co-clustered with CIBN-mCh-CaMKII WT indicating these proteins were able to interact

Video S2. Monitoring of light induced co-clustering of GFP-CaMKII R396st with CIBN-mCh-CaMKII WT and untagged-Cry2olig expressed in HEK293 cells, related to Figure 2 Following blue-light stimulation, GFP-CaMKII R396st failed to co-cluster with CIBN-mCh CaMKII WT indicating this CaMKII variant was unable to interact with CaMKII WT and suggesting that the R396st mutation results in decreased subunit oligomerization

**Figure 3:**
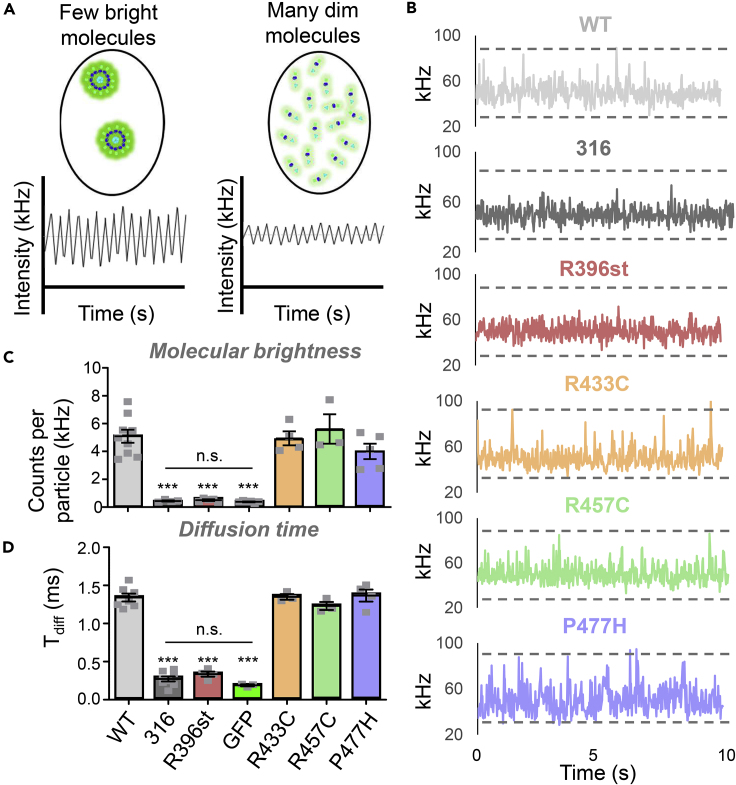
Analysis of CaMKII variant oligomeric state with fluorescence correlation spectroscopy GFP-tagged CaMKII and variants were expressed in and extracted from HEK293 cells and analyzed for molecular brightness by fluorescence correlation spectroscopy. (A) Confocal microscopy is used to monitor diffusion of fluorescently tagged molecules in and out of the focal volume. The fluctuations seen in the average fluorescence intensity are characteristic of the intrinsic brightness of the molecule being studied. Molecular brightness is proportional to the number of fluorophores in an independently diffusing molecule, which, in turn, is proportional to the oligomeric state of that molecule. (B) Representative fluorescence intensity traces. Dotted black lines indicate the minimum and maximum fluorescence values from the WT CaMKII trace. 316 is a CaMKII truncation mutant that completely lacks the association domain. (C) Molecular brightness determined by FCS. ∗∗∗p<0.001 vs WT; n.s., not significant. (D) Diffusion time determined by FCS; ∗∗∗p<0.001 vs WT; n.s., not significant. Data in (C) and (D) are represented as mean ± SEM; statistical analysis was by one-way ANOVA with Tukey's post hoc analysis.

While the LINC assay showed that all CaMKII association domain variants (except R396st) interact with WT CaMKII, this assay did not address whether variant subunits interact with other variant subunits of the same type. The effect of the mutations on holoenzyme formation was further tested by fluorescence correlation spectroscopy (FCS) and molecular brightness analysis of CaMKII variants that were expressed individually. This assay has been developed by Dr. Steven Vogel's group for the analysis of CaMKII multimerization (Sarkar et al., 2017), and our experiments yielded very similar results for both molecular brightness and diffusion constant, both for full-length CaMKII wild type and for the 316 mutant that completely lacks the association domain (Figure 3). Molecular brightness is proportional to the number of fluorescently tagged subunits in an independently diffusing unit (Figure 3A). The molecular brightness of R396stop was significantly decreased compared to WT 12-meric CaMKII but not significantly different than that of soluble GFP or truncated CaMKII (316) that completely lacks the association domain (Figures 3B and 3C), indicating that this variant is monomeric. By contrast, the association domain missense variants R433C, R457C, and P477H were all indistinguishable from the 12-meric WT holoenzyme. The same conclusion can be reached from the measurements of the diffusion time constants (Figure 3D), which were significantly faster than WT for the R396st variant but not for the other variants; in fact, the R396st variant was indistinguishable from the monomeric 316 mutant and from unconjugated GFP (Figure 3D). Additionally, R396st showed some nuclear localization by both live imaging of GFP-CaMKII^R396st^ and immunostaining of untagged CaMKII^R396st^ (Figure S1), further supporting that this variant is monomeric. Together, these results indicate (i) that the association domain missense mutations do not have dramatic effects on the CaMKII holoenzyme formation but (ii) that the R396stop mutation completely disrupts the holoenzyme and makes the kinase monomeric.

### Association domain stop mutation impairs GluN2B binding

**Figure 4:**
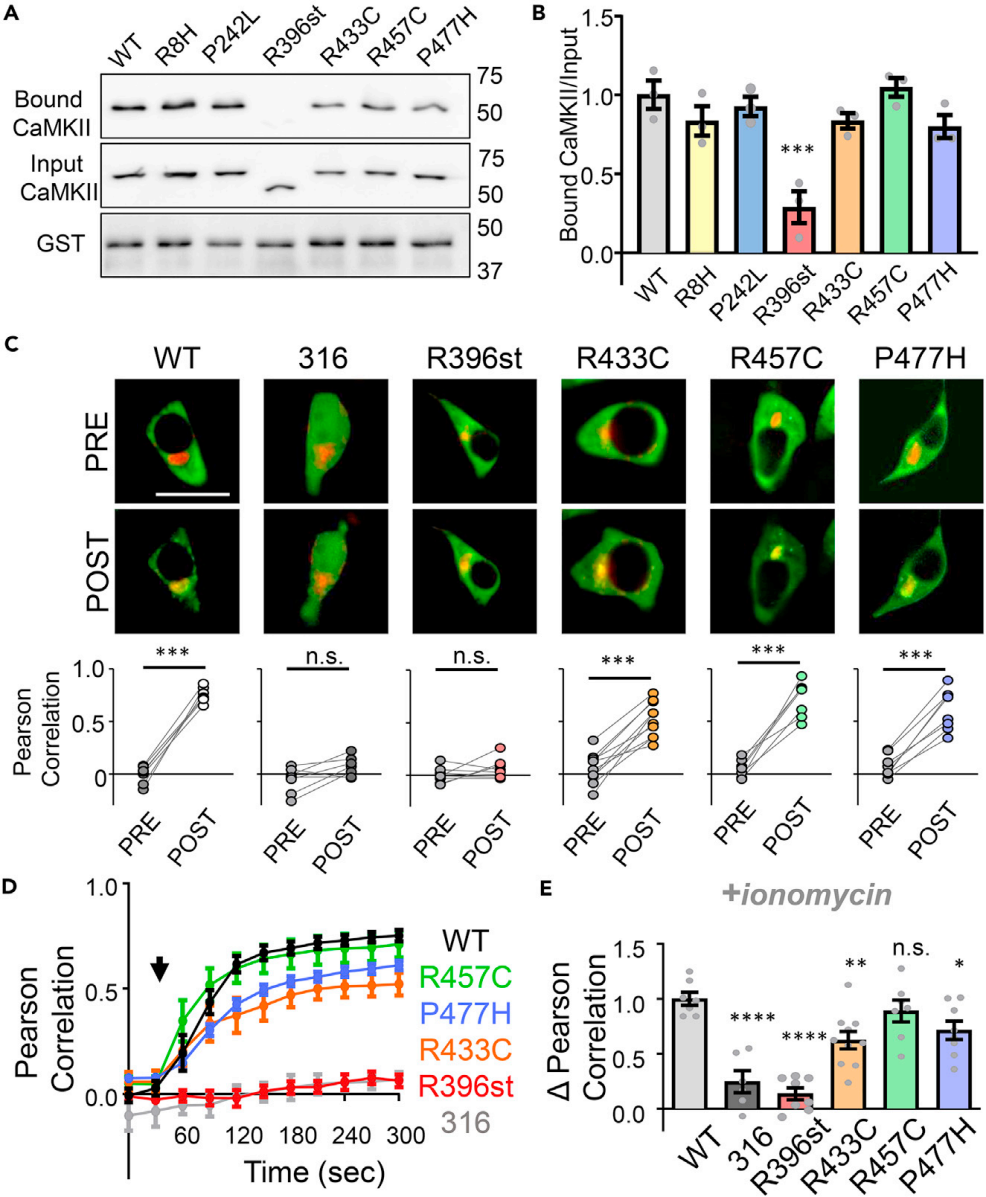
Effects of CaMKII mutations on CaMKII-GluN2B binding Data in (B), (D), and (E) are represented as mean ± SEM and statistical analysis was by one-way ANOVA with Tukey's post hoc analysis unless indicated otherwise. (A) Representative immunoblots from *in vitro* binding assays of CaMKII and its variants to GST-GluN2B immobilized on microtiter plates. (B) Quantitation of the binding assays show significantly reduced GluN2B binding only for the R396st variant; ∗∗∗p<0.001 versus WT. (C) Co-localization of GFP-CaMKII (green) with a membrane-targeted mCherry-GluN2B C-tail (red) in HEK293 cells is enhanced by ionomycin-induced Ca^2+^ signals. Shown are representative images pre- and 5 min post-ionomycin, as well as paired quantitation of co-localization; ∗∗∗p<0.001 in paired Student's t-test. 316 is a CaMKII truncation mutant that completely lacks the association domain. Scale bar = 10μm. (D) Time course of CaMKII co-localization with GluN2B in HEK cells. The black arrow indicates the time point of ionomycin treatment. (E) Net change in CaMKII/GluN2B co-localization after ionomycin treatment (t = 5min – t = 0min); ∗p<0.05; ∗∗p<0.01; ∗∗∗∗p<0.0001; n.s., not significant versus WT.

CaMKII binding to GluN2B is mediated by the kinase domain but also requires the holoenzyme (Bayer et al., 2006; Strack et al., 2000), likely by enabling an avidity effect allowed by simultaneous binding of multiple kinase domains. Thus, we first tested both kinase and association domain variants for GluN2B binding in our established *in vitro* assay that utilizes the immobilized GST-GluN2B C-terminus (Buonarati et al., 2020; Goodell et al., 2017). All binding reactions contained Ca^2+^/CaM, which is required to initiate the binding. All missense mutations in the kinase or association domain showed normal binding; impaired binding was observed only with the R396st variant (Figures 4A and 4B).

Next, we tested the effects of the association domain mutations on GluN2B binding in live cells in our established HEK293 cell co-localization assay (Tullis et al., 2020). Briefly, CaMKII variants were co-expressed in HEK293 cells with a membrane-targeted GluN2B intracellular C-tail. Images were collected before and up to 5 min after stimulation with the Ca^2+^ ionophore, ionomycin. WT CaMKII, R457C, R433C, and P477H rapidly co-localized with GluN2B after stimulation (Figures 4C and 4D; Video S3). The truncated R396st variant, however, completely failed to co-localize with GluN2B (Figures 4C–4E; Video S4), similar to the monomeric 316 mutant. These data indicate that the R396st mutation causes CaMKII functional impairment and support the conclusion that the 12-meric holoenzyme is required for Glu2NB binding.

Video S3. Monitoring of GFP-CaMKII WT binding to mCh-GluN2b c-tail in HEK293 cells, related to Figure 4 Following calcium stimulation using ionomycin, CaMKII WT was able to co-localize with mCh-GluN2b indicating CaMKII WT and GluN2b are able to bind

Video S4. Monitoring of GFP-CaMKII R396st binding to mCh-GluN2b c-tail in HEK293 cells, related to Figure 4 Following calcium stimulation using ionomycin, CaMKII R396st did not co-localize with mCh-GluN2b indicating the R396st mutation disrupts CaMKII-GluN2b binding

### Association domain stop mutation impairs synaptic enrichment

**Figure 5:**
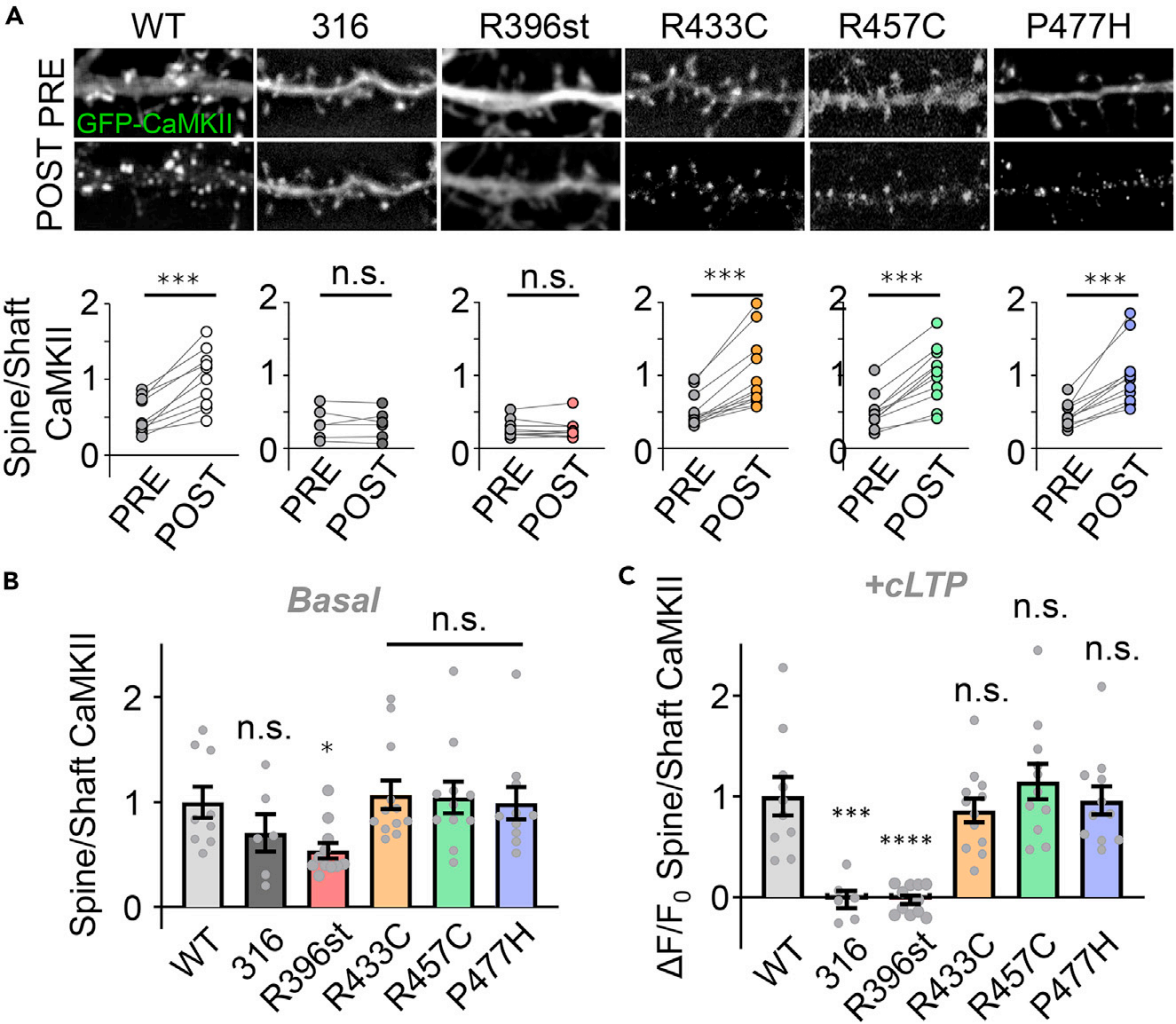
Effects of CaMKII association domain mutations on cLTP-induced synaptic targeting Data in (B) and (C) are represented as mean ± SEM and statistical analysis was by one-way ANOVA with Tukey's post hoc analysis unless indicated otherwise. (A) Representative images and quantification of CaMKII enrichment at excitatory synapses on dendritic spines of rat neurons (DIV 14) in response to cLTP stimuli (1 min 100 μM glutamate/10 μM glycine); ∗∗∗p<0.001; n.s. p>0.05 in paired Student's t-test. Scale bar = 10μm. (B) Quantitation of basal CaMKII spine localization. ∗p<0.05; n.s., not significant versus WT. (C) cLTP induced synaptic CaMKII targeting but not for the 316 mutant or the R396st variant; ∗∗∗p<0.001, ∗∗∗∗p<0.0001, or n.s., not significant versus WT. 316 is a CaMKII truncation mutant that completely lacks the association domain.

GluN2B binding is thought to mediate synaptic targeting of CaMKII during LTP (Bayer et al., 2001; Halt et al., 2012). We determined whether deficiencies in our *in vitro* and HEK293 cell GluN2B binding assays are reflected by impaired synaptic accumulation of CaMKII in cultured neurons. In hippocampal neurons, we monitored the movement of CaMKII WT versus variants in response to chemical LTP stimuli (cLTP; 1 min after 100 μM glutamate, 10 μM glycine). Excitatory synapses were identified by morphology (on dendritic spines) and by co-transfection with an intrabody against the excitatory marker protein PSD-95 (Cook et al., 2019; Gross et al., 2013). WT, R433C, R457C, and P477H CaMKII all significantly accumulated at excitatory synapses to a similar extent after cLTP (Figures 5A and 5B; Video S5). By contrast, R396st failed to accumulate at spines after stimulation and additionally showed significantly decreased basal synaptic enrichment (Figures 5A–5C; Video S6). Together, these data show that the monomeric CaMKII R396 variant that is unable to bind GluN2B in HEK cells or *in vitro* is also impaired for accumulation at excitatory synapses.

Video S5. Monitoring of GFP-CaMKII WT synaptic translocation in cultured rat hippocampal neurons, related to Figure 5 Following cLTP stimulation, GFP-CaMKII WT targets dendritic spines which house excitatory synapses

Video S6. Monitoring of GFP-CaMKII R396st synaptic translocation in cultured rat hippocampal neurons, related to Figure 5 Following cLTP stimulation, GFP-CaMKII R396st does not target dendritic spines indicating the R396st mutation impairs cLTP-induced synaptic targeting

### Two of the mutations impair CaMKII autophosphorylation at T286, and one mildly affects stimulated kinase activity

**Figure 6:**
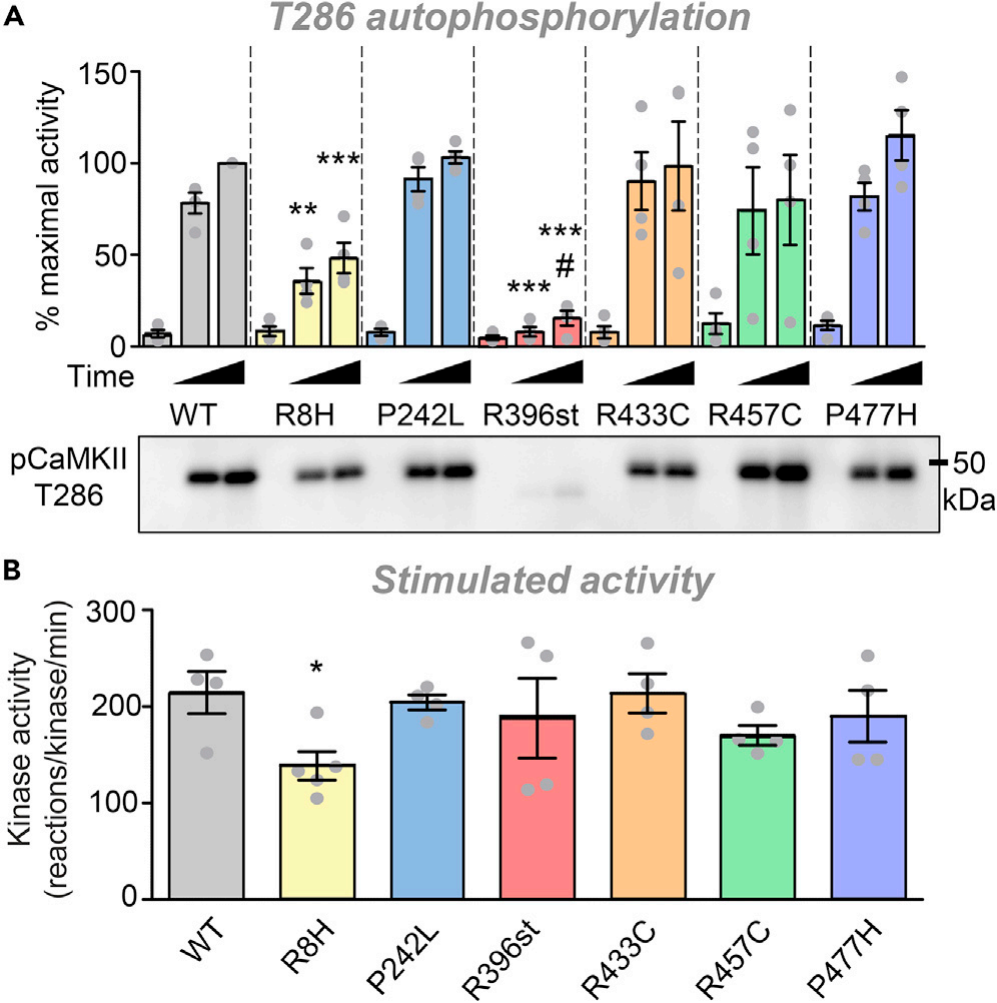
Effect of the CaMKII mutations on T286 autophosphorylation and kinase activity *in vitro* Data are represented as mean ± SEM and statistical analysis was by one-way ANOVA with Tukey's post hoc analysis. (A) CaMKII T286 autophosphorylation was detected by western blot and quantified after increasing reaction times (0, 15, 60 s). No differences were detected at the 0 s control time point. At both 15 and 60 s, autophosphorylation was significantly reduced only for the R8H and the R396st variants; ∗∗p<0.01, ∗∗∗p<0.01 in one-way ANOVA for each reaction time, compared to WT. The minimal apparent phospho T286 increase for the R396st variant was statistically significant only for the longer time point and only by t test (#p = 0.045) and not by ANOVA. (B) Ca^2+^/CaM-stimulated CaMKII activity was measured by incorporation of 32P into the substrate peptide syntide 2. Significantly reduced activity was detected only for the R8H variant; ∗p<0.05 in one-way ANOVA with Bonferroni comparison to WT.

CaMKII autophosphorylation at T286 is a major regulatory feature of CaMKII, as it generates Ca^2+^-independent “autonomous” kinase activity and is required for both LTP, which is studied here, and LTD, an opposing form of plasticity (Cook et al., 2021; Coultrap et al., 2014a, 2014b). Like GluN2B binding, this T286 autophosphorylation could be affected by mutations in either the kinase or association domain: Any phosphorylation reaction obviously requires the kinase domain, but T286 autophosphorylation additionally requires the holoenzyme structure, as it occurs as a trans-subunit reaction between two subunits within a holoenzyme. Thus, we tested both kinase and association domain variants for potential impairments in T286 autophosphorylation using our established *in vitro* assay (Cook et al., 2021). As expected, based on the effect on holoenzyme structure, T286 autophosphorylation was significantly impaired by the monomeric R396st variant (Figure 6A) but not by the other association domain variants that had no dramatic effect on holoenzyme formation. Additionally, T286 autophosphorylation was impaired by the R8H but not the P242L mutation in the kinase domain (Figure 6A). For both the R396st and the R8H stop mutation, the impairment in T286 autophosphorylation was evident, both after 15 and 60 s reaction times (Figure 6A).

Next, we decided to test the effect of the mutations on Ca^2+^/CaM-stimulated kinase activity with our established CaMKII assay that utilizes incorporation of ^32^P into substrate peptides *in vitro* (Coultrap et al., 2010). As expected, all association domain variants showed normal CaMKII activity (Figure 6B). For the kinase domain variants, a mild reduction in activity was observed only for the R8H but not the P242L variant (Figure 6B). This is consistent with the reduced T286 autophosphorylation by only R8H kinase domain mutation.

### The CaMKII mutations do not reduce CaMKII expression

**Figure 7:**
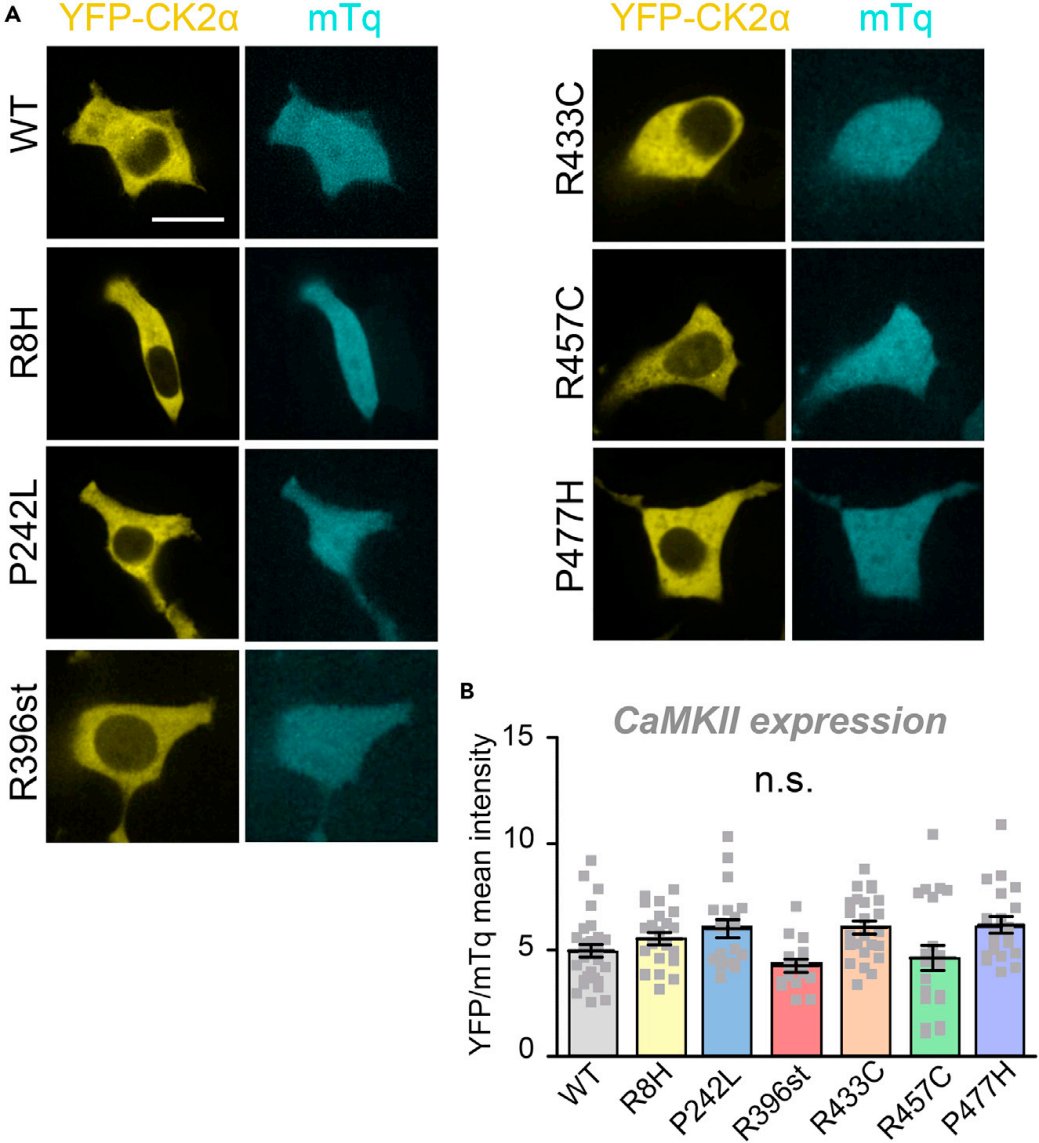
Expression of CaMKII variants in HEK293 cells (A) Representative images of mTq-IRES-YFP2-CaMKII (CK2α) WT and variant expression in HEK293 cells. Scale bar = 10 μm. (B) Quantitation of YFP corrected for mTq expression indicated no effect on the mutations on CaMKII expression levels; n.s., not significant in one-way ANOVA with Tukey's post hoc analysis. Data are represented as mean ± SEM.

As only two of the six CaMKII mutations from patients with schizophrenia showed obvious impairments in CaMKII function, we decided to additionally test the effect of the mutations on expression levels. This rationale was further enhanced by the previous description that heterozygous CaMKIIα knockout mice have a schizophrenia-related phenotype (Frankland et al., 2008; Yamasaki et al., 2008). To test the expression levels of schizophrenia variants, we created an IRES-containing bicistronic vector (mTurquoise-IRES-YFPCaMKIIα) encoding YFP-CaMKII and soluble mTurquoise as a standard for ratiometric quantification. None of the mutations resulted in decreased CaMKII expression in a human cell line, HEK293 (Figure 7).

## Discussion

Together, the results presented here show impaired biochemical functions of two CaMKII variants found in patients with schizophrenia. Notably, the truncated R396st mutation had impaired (i) holoenzyme formation (both when expressed alone or co-expressed with WT CaMKII), (ii) GluN2B binding *in vitro* and in HEK293 cells, (iii) synaptic targeting in hippocampal neurons, and (iv) *in vitro* T286 autophosphorylation. The R8H kinase domain variant had significantly impaired T286 autophosphorylation and mildly reduced substrate phosphorylation. The remaining four mutations did not show obvious impairments in CaMKII function or expression in the experiments performed here, highlighting that the presence of a mutation in a disease population does not necessarily mean that the mutation is a primary driver of disease. Indeed, two of these mutations have now been detected also in the general population (P242L and R457C; see Table S1), and this may be the case in the future also for the other two mutations for which no obvious effects were observed here (R433C and P477H). However, at least for the R396st mutation, the severity of the observed effects should strongly predict a severe neurological phenotype. Indeed, neurological phenotypes have been described for several other CaMKII mutations with similar or less severe biochemical effects, including (i) E183V, which disrupts T286 autophosphorylation and is linked to autism spectrum disorder (Kury et al., 2017; Stephenson et al., 2017), and (ii) P212Q, which causes increased T286 autophosphorylation and is associated with seizures (Akita et al., 2018; Kury et al., 2017). Notably, patients were heterozygous for R396st as well as for E183V and P212Q. In mice, heterozygous knockout was sufficient to elicit a schizophrenia-related phenotype (Yamasaki et al., 2008), and heterozygous T286A mutation (which prevents the regulatory autophosphorylation at T286 that has been reduced also by the R396st and R8H mutations studied here) is sufficient to interfere with extinction learning (Kimura et al., 2008).

Clearly, CaMKII mutation is not a major genetic driver in the vast majority of schizophrenia cases. However, this does not rule out a general involvement of CaMKII dysregulation or a potential for CaMKII as a therapeutic target in schizophrenia. Indeed, our findings suggest that a severely dysregulating CaMKII mutation can be a driver for schizophrenia in humans. This finding provides important complement to several studies in rodents: Heterozygous CaMKII knockout directly causes a schizophrenia-related phenotype in mice (Yamasaki et al., 2008), and CaMKII misregulation has been implicated by several rodent models of schizophrenia. For instance, neonatal ventral hippocampus (NVH) lesion models of schizophrenia result in both decreased CaMKII activity and cognitive impairment (Yabuki et al., 2013a). Ketamine models of schizophrenia have decreased total CaMKII expression (Ogundele and Lee, 2018). Furthermore, preclinical studies have shown promise in CaMKII-targeted therapies for the treatment of cognitive dysfunction in schizophrenia: CaMKII activation by ST101 in an NVH lesion model of schizophrenia enhanced cognition (Yabuki et al., 2019). By contrast, currently used antipsychotics do not benefit cognitive defects, neither in patients with schizophrenia nor in the NVH lesion model (Yabuki et al., 2013a). Importantly, CaMKII is well established as a central mediator for forms of synaptic plasticity thought to underlie cognition (Bayer and Schulman, 2019), further enhancing plausibility of CaMKII, a pharmacological target for restoring this major unmet need in schizophrenia therapy.

The R396st, R433C, R457C, and P477H mutations are all found in the CaMKII association domain, which is required for holoenzyme formation, but only the R396st variant was found to impair holoenzyme formation. Indeed, all of the missense mutations are localized to the outer surface of the association domain (see Figure 1) and would not be expected to directly impair association of subunits. By contrast, the R396st mutation deletes a significant part of the association domain. For R396st, the impaired holoenzyme formation is most likely the direct cause for all of its other impairments found here.

T286 autophosphorylation occurs by a trans-subunit mechanism, making this reaction impossible in monomers. GluN2B binding is ablated with CaMKII monomers (Strack et al., 2000), likely due to avidity effects that require full holoenzymes. As GluN2B binding mediates synaptic accumulation of CaMKII during LTP (Bayer et al., 2001; Halt et al., 2012), the reduced synaptic enrichment of R396st after cLTP stimulation was expected. Additionally, we also found decreased basal enrichment of R396st in dendritic spines, similarly as we have described previously for another mutation that impairs GluN2B binding (Tullis et al., 2020). Thus, the decreased basal synaptic localization of the R396st variant could be due to impaired basal GluN2B binding. Alternatively, it could be due to impaired binding to other synaptic proteins, such as densin-180 (Walikonis et al., 2001). Indeed, CaMKII binding to densin-180 requires the association domain and is enhanced by T286 autophosphorylation that is impaired by the R396st mutation (Walikonis et al., 2001). Notably, GluN2B variants have also been identified in patients with schizophrenia (Myers et al., 2019), and deletion of densin-180 causes a schizophrenia-related phenotype in mice (Carlisle et al., 2011).

The decreased T286 autophosphorylation and stimulated activity seen with the R8H variant were somewhat unexpected, as the mutation does not occur in the active site of the kinase. Decreased T286 phosphorylation is in line with a previous study that showed decreased T286 autophosphorylation in an NVH lesion model of schizophrenia (Yabuki et al., 2013b), although this is not found in all rodent schizophrenia models (Ogundele and Lee, 2018; Musazzi et al., 2011). Our current study showed decreased T286 autophosphorylation for both R8H and R396st mutations, but it is unclear if this decrease is a common feature in patients with schizophrenia.

Our findings here elucidate functional impairments of two patients with schizophrenia-derived CaMKII mutations on holoenzyme formation, GluN2B binding, synaptic targeting, and kinase activity and provide insight into the role of CaMKII dysregulation in neuropsychiatric disease. Given the plausibility of CaMKII as a driver for neurological phenotypes, it will be interesting to elucidate if mutations in other genes that increase the genetic risk for schizophrenia also cause dysregulation of CaMKII, even if indirectly.

### Limitations of the study

Characterization of the biochemical effects of mutations found in patients can provide important new insight into underlying disease mechanisms. However, the occurrence of a mutation in a patient does not mean that the mutation is driving the disease. Additionally, no array of characterization can test for all possible effects of a mutation. For instance, we investigated the CaMKII variants for effects on binding to GluN2B because this binding (i) is thought to require holoenzyme assembly and (ii) is known to be required for normal LTP and synaptic CaMKII accumulation. However, this does not mean that GluN2B binding is the only synaptic CaMKII protein interaction with potential functional relevance (for review see Bayer and Schulman, 2019; Colbran, 2004; Hell, 2014). Also, while we tested the CaMKII variants for effects on maximal GluN2B binding and on maximal stimulated kinase activity, we did not test for potential effects on the Ca^2+^/CaM-sensitivity for the induction of these two processes. Sensitivity to activation by Ca^2+^/CaM could be a potentially relevant parameter but would appear more likely for mutations within the regulatory domain and less likely for the mutations in the kinase or association domains that were characterized here. Additionally, accurate detection of differences in Ca^2+^/CaM-sensitivity is not very amenable for screening of multiple variants. However, for the two variants for which we did not detect any biochemical effects here and which have not yet been detected in the general population (R433C and P477H), further analysis of Ca^2+^/CaM-sensitivity and binding to other proteins would be strongly indicated if they are detected in additional patients with schizophrenia in the future.

## STAR★Methods

### Key resources table

**Table:** 

REAGENT or RESOURCE	SOURCE	IDENTIFIER
**Antibodies**		

CaMKIIα	Made in House	CBα2; RRID: AB_2533032
CaMKIIα pT286	Phospho-Solutions	p1005-286; RRID: AB_2492051
GST	Millipore	AB3282; RRID: AB_91439
Goat anti-Rabbit	GE Healthcare	NA934V; RRID: AB_772206
Goat anti-Mouse	GE Healthcare	NA931V; RRID: AB_772210

**Chemicals, peptides, and recombinant proteins**		

Papain	Worthington	LS 03126
Lipofectamine 2000	Invitrogen	11668027
B-27 supplement	Gibco	17504044
Glutamate	Sigma	6106-04-3
Glycine	Sigma	56-40-6
cOmplete protease inhibitor cocktail (EDTA-free)	Roche	1187380001
Microcystin-LR	Calbiochem	475815
Glutathione-coated magnetic beads	Pierce	78601
Calmodulin	Made in house	CaM
Ca^2+^/CaM-dependent kinase II α	Made in house	CaMKII
GST-GluN2B C-tail	Made in house	GST-GluN2Bc
Fluorescein	Sigma	F2456-2.5G

**Critical commercial assays**		

SuperSignal West Femto	Thermo Fisher	34095

**Deposited data**		

Raw and analyzed data	This paper	Mendeley Data, V1, doi: 10.17632/t5zhhhrfb9.1

**Experimental models: cell lines**		

Primary hippocampal cultures	Laboratory of K. Ulrich Bayer	N/A
HEK293 cells	ATCC	CRL-1573

**Experimental models: organisms/strains**		

Rat: Sprague-Dawley	Charles River Labs	N/A

**Software and algorithms**		
Prism 7.0	Graphpad	RRID: SCR_002798
AlphaEase FC 4.0	Alpha Innotech	N/A
ImageJ	NIH	RRID:SCR_003070
Slidebook 6.0	Intelligent Imaging Innovations (3i)	RRID:SCR_014300
Pycorrfit 1.1.7	(Muller et al., 2014)	N/A

### Resource availability

#### Lead contact

The Lead Contact is K. Ulrich Bayer (ulli.bayer@cuanschutz.edu).

#### Materials availability

Requests for resources, reagents, and further information should be directed to and will be fulfilled by the lead contact. This study did not generate new unique reagents, other than the mTurquoise2-IRES-YPF2-CaMKIIα vector for expression analysis..

### Experimental model and subject details

All animal treatment was approved by the University of Colorado Institutional Animal Care and Use Committee (IACUC), in accordance with NIH guidelines. Animals are housed at the Animal Resource Center at the University of Colorado Anschutz Medical Campus (Aurora, CO) and are regularly monitored with respect to general health, cage changes, and overcrowding. All animals were provided with *ad libitum* access to food and water and were housed in standard cages on a 12/12 light/dark schedule. No experiments were conducted on living animals. The day of birth is defined as postnatal day (p) 0. E14 Sprague Dawley rat dams were ordered from Charles River. Upon birth, p0-1 mixed sex pups were used for primary hippocampal neuronal cultures. Cultures were imaged on DIV 14-17.

### Method details

#### Materials and DNA constructs

All cell culture reagents were obtained from Invitrogen and all chemicals were obtained from Sigma, unless indicated otherwise. GFP-CaMKII constructs, CIBN-mCherry-CaMKII constructs (Bayer et al., 2006; Cook et al., 2018; Taslimi et al., 2014), and mCh-GluN2b constructs (Goodell et al., 2016) were here further modified as indicated. The mTurquoise2-IRES-SYPF2-CaMKIIα vector was constructed from an mCherry-C1 vector with an internal ribosomal entry site (IRES) inserted in the multi-cloning site (gift from Dr. Matthew Kennedy). mCherry was removed by cleavage with NheI and BsrGI followed by gel purification. mTurquoise was PCR amplified with Phusion HF polymerase (ThermoFisher) from pmTurquoise2-C1 vector using the primers: 5’-CAAGGGTACCGCAATACCGGAGTACTACTTGTACAGCTCGTCCATGCCGAG-3’ and 5’-ATCCGCTAGCGCTACCGGTCGCCACCATGGTGAGCAAGGGCGAGGAGCTGTTC-3’. The PCR product was then cleaved with NheI and BsrGI then ligated into the IRES vector to replace mCherry. SYFP2-CaMKIIalpha was PCR amplified from an existing vector with the primers with primers 5’-ATCAAGCTTATCGATACCGTCGACCTCGAGCCACCATGGTGAGCAAGGGCGAGGAGCTGT-3’ and 5’-AGTTATCTAGATCCGGTGGATCCCGGGCTTAAGTCAATGGGGCAGGACGGAGGGCGCCCC-3’. The IRES vector was cleaved with XhoI and XmaI, then the SYFP2-CaMKIIα PCR product was inserted into the MCS by Gibson assembly of the above mTurquoise2-IRES vector using the NEBuilder kit (NEB). The resulting vector contains CMVpromoter-mTurquoise2-IRES-SYFP2-CaMKIIα.

#### HEK293 cell culture

HEK293 cells were maintained in Dulbecco’s modified Eagle’s medium supplemented with fetal bovine serum and penicillin/streptomycin.

#### Primary rat hippocampal culture

Primary dissociated hippocampal neuron cultures were prepared from p0-1 mixed sex Sprague Dawley rat pups (Cook et al., 2019). Neurons were plated at medium density on poly-D-lysine and laminin coated coverslips in neurobasal A supplemented with B27 and l-glutamine and incubated at 37°C with 5% CO_2_. Additional cell division was prevented with 70 mM 5-fluoro-29-deoxyuridine plus 140 mM uridine at 4-5 days *in vitro* (DIV) and neurons were fed at 5–6 DIV by replacing half of the culture media. Transfections using Lipofectamine 2000 (Invitrogen) were performed at DIV 12–13 and neurons were imaged DIV 13–14.

#### Imaging acquisition

Microscopic imaging for Figures 2, 4, and 5 was performed using a 100 × 1.4NA objective on a Zeiss Axiovert 200 M (Carl Zeiss GmbH, Oberkochen, Germany) fitted with a 63× objective (1.4NA, planApo). and controlled by SlideBook software (Intelligent Imaging Innovations, Denver, CO). Imaging for Figure7 was performed using an Axio Observer microscope (Carl Zeiss) fitted with a 63× Plan-Apo/1.4 numerical aperture objective, using 445-, and 515 laser excitation and a CSU-XI spinning disk confocal scan head (Yokogawa) coupled to an Evolve 512 EM-CCD camera (Photometrics). All imaging analysis was completed using SlideBook software. All representative images were prepared using Fiji software (ImageJ, NIH). For all imaging experiments, focal plane z stacks (0.3-μm steps; over 1.8–2.4 μm) were acquired and deconvolved to reduce out-of-focus light. 2D maximum intensity projection images were then generated and analyzed by an experimenter blinded to experimental conditions.

#### CaMKII live imaging in HEK293 cells

HEK293 cells were plated on No. 1 coverslips and transfected by the calcium phosphate method (Tullis et al., 2020). A 1:6 ratio of GFP-labeled CaMKIIα (WT, 316, R396st, R433C, R457C, or P477H) and mCh-GluN2B c-tail and allowed to express for 16–24 hours. Cells were imaged every 30 seconds over 6 min and stimulated with ionomycin (10 μM) at 1 min. A Pearson’s correlation of fluorescent overlap for each time point was calculated using hand drawn masks encompassing the entire cell and excluding the nucleus. Raw Pearson’s correlations are shown for starting values. Sample size is indicated by individual data points on graph and statistical analysis is detailed in figure legends.

#### Light induced co-clustering (LINC) assay

Live imaging of HEK293 cells was carried out at 32°C in HEPES-buffered imaging solution containing (in mM): 130 NaCl, 5 KCl, 10 HEPES (pH 7.4), 20 glucose, 2 CaCl_2_, and 1 MgCl_2_. LINC assay was performed with various CaMKII mutant constructs (Cook et al., 2018). Briefly, HEK293 cells were transiently transfected by the calcium phosphate method as previously described (Bayer et al., 2001, 2006) using a 1:1:1 ratio of GFP-labeled CaMKIIα (WT, 316, R396st, R433C, R457C, or P477H), CIBN-mCh-labeled bait WT CaMKII and unlabeled CRY2olig (Taslimi et al., 2014). 24 h after transfection, transfected cells were identified using 488 nm blue light to activate CIBN-CRY2olig clustering, and images were acquired 1 minute after the initial blue-light exposure. Clustering of GFP-CaMKII WT and variants with mCh-CIBN-WT CaMKII was quantified via clustering index (GFP/mCh ratio in clusters vs. non-clustered regions). Clusters were identified using a threshold mask of the CIBN-mCh fluorescence intensity (mean + 2 standard deviations of whole cell mCh intensity) for each cell. Non-clustered regions were characterized by subtracting the mean fluorescence intensity of the clusters from the intensity of the whole cell. Mean GFP and mCh background intensity was subtracted from each cell prior to quantifying the clustering index. Sample size is indicated by individual data points on graph and statistical analysis is detailed in figure legends.

#### mTurquoise-IRES-YFP2-CaMKII expression vector analysis

HEK293 cells were transfected by the calcium phosphate method and imaged as described in the previous section (LINC assay). A cytoplasmic mask was hand drawn on each cell and used to calculate the integrated density for YFP and mTurquoise (mTq). Total CaMKII expression was quantified by the ratio of YFP fluorescence divided by mTq fluorescence. Sample size is indicated by individual data points on graph and statistical analysis is detailed in figure legends.

#### Live imaging of cultured hippocampal neurons

Live imaging of cultured rat hippocampal neurons occurred in a climate-controlled chamber at 34°C in a HEPES buffered aCSF solution containing the following (in mM): 130 NaCl, 5 KCl, 10 HEPES pH 7.4, 20 glucose, 2 CaCl_2_, and 1 MgCl_2_ (adjusted to proper osmolarity with sucrose). Chemical LTP (cLTP) was induced using 100 μM glutamate and 10 μM glycine. After 1 min of cLTP treatment, 5 volumes of fresh aCSF were perfused through the imaging chamber to wash out the stimulation. This cLTP stimulation has been shown to induce robust CaMKII accumulation in spines (Barcomb et al., 2013; Cook et al., 2019). Images were quantified using the starting image and image acquired 1 minute post washout. Spine-to-shaft ratio was calculated as background-subtracted mean intensity of circular regions of interests (ROIs) of at least 15 dendritic spines divided by the mean intensity of a line drawn in the corresponding dendritic shaft. Spines were identified by morphology and by expression of an intrabody against a marker protein for excitatory synapses, PSD-95 (Cook et al., 2019; Gross et al., 2013). Changes in CaMKII localization were determined by dividing the net change in spine-to-shaft ratio by the starting spine-to-shaft ratio (Change in fluorescence divided by starting fluorescence, ΔF/F0 Spine/Shaft CaMKII). Sample size is indicated by individual data points on graph and statistical analysis is detailed in figure legends.

#### Protein preparation

CaMKIIα WT and variants (316, R396st, R433C, R457C, or P477H) were expressed in HEK293 celsl sly (Coultrap and Bayer, 2014). HEK293 cells were split and grown to 50% confluency prior to transfection with CaMKII constructs using the Ca^2+^/phosphate method. Constructs were expressed 16–24 hours prior to protein extraction. Media was removed and replaced with ice cold Dulbecco’s phosphate buffered saline without CaCl_2_ or MgCl_2_ and cells were scraped off with a rubber cell scraper and spun at 1000xg for 5 min. Supernatant was discarded and pellets were resuspended in homogenate buffer containing (in mM) 50 PIPES pH 7.12, 1 EGTA, 1 DTT, 10% glycerol and complete protease inhibitor cocktail (Roche). Cells were spun briefly at 16,000xg to re-pellet and lysed with a motorized pellet homogenizer (Kontes). Homogenate was then spun at 16000xg for 30 min to remove cell debris and resulting supernatant was collected for use in biochemistry experiments.

GST-GluN2Bc contains the cytoplasmic C-terminal tail of GluN2B (amino acids 1,122–1,482) and was purified after bacterial expression (Bayer et al., 2001).

#### *In vitro* CaMKII activity and autophosphorylation

Kinase reactions (1 min at 30°C) were performed essentially as described (Coultrap and Bayer, 2011). Briefly, wild type or variant CaMKII (2.5 nM subunits, unless stated otherwise) was added to a mix of 50 mM PIPES pH 7.2, 0.1% BSA, 1 μM CaM, 1 mM CaCl_2_, 10 mM MgCl_2_, 100 μM [γ-32P]ATP (∼1 Ci/mmole), 75 μM substrate peptide (syntide 2, or as indicated), and inhibitor peptide as indicated. Reactions with 0.1 nM CaMKII were done for 10 min. For all kinase activity assays, reactions were stopped by spotting onto P81 cation exchange chromatography paper (Whatman) squares. After extensive washes in water, phosphorylation of the substrate peptide bound to the P81 paper was measured by the Cherenkov method.

For analysis of T286 autophosphorylation, HEK cell extracts containing wild type or variant CaMKII (30 nM subunits) was added to an ice cold mix of 50 mM PIPES pH 7.2, 0.1% BSA, 2 μM CaM, 2 mM CaCl_2_, 10 mM MgCl_2_, 1 mM ATP. Reactions were performed on ice for 0, 15 or 60 seconds then stopped by addition of of gel loading buffer and boiling for 5 minutes. Phospho-T286 was measured by Western blot. Sample size is indicated by individual data points on graph and statistical analysis is detailed in figure legends.

#### CaMKII-GluN2B binding *in vitro*

CaMKII binding to GluN2B was tested with GST-GluN2Bc (Glutathione S-transferase tagged to the GluN2B cytoplasmic C-terminus, amino acids 1,120–1,482) immuno-immobilized in Glutathione-coated microtiter plate wells (Pierce) (Barcomb et al., 2013; Goodell et al., 2014). Briefly, plates were washed 4 times in wash buffer (PST) containing 50 mM PIPES pH 7.12, 150 mM NaCl, and 0.05% Tween-20. GST-GluN2Bc (300 nM/well, diluted in PST containing 0.05 % BSA) was allowed to bind to plate wells for one hour under gentle agitation at room temperature and washed three times with PST. Plate wells were then blocked in 5% BSA in PST for 1 hour under gentle agitation at room temperature and washed 1 time with PST following the removal of the blocking solution. HEK293 extracts containing CaMKII wild type or variants (at 40 nM) was bound for 30 min in CaMKII binding buffer (1:1 in 100 μl) containing 50 mM PIPES pH 7.12, 150 mM NaCl, 9.5 mM MgCl_2_, 100 μM ATP, 0.05% BSA, and 0.05% Tween-20 and Ca^2+^/CaM (2 mM/1 μM). This binding reaction was incubated for 15 min under gentle agitation at room temperature. Unbound CaMKIIα was discarded, and plate wells were washed in PST containing 1 mM EGTA 4 times to remove remnants of high concentration molecular crowding agents. 60 μl of loading buffer containing 2% SDS was then added to the wells and samples were incubated at 95°C for 10 minutes to dissociate bound proteins from the plates. Samples were then analyzed by SDS-PAGE and Western analysis. Sample size is indicated by individual data points on graph and statistical analysis is detailed in figure legends.

#### Immunoblot analysis

For immunoblot analysis, protein was transfered onto PVDF membrane (Goodell et al., 2017) and detected using antibodies specific for CaMKIIα (CBα2) or GST. Briefly, 10 μg of total protein was subjected to SDS-PAGE on 7.5% polyacrylamide gels made in house and transferred to PVDF membrane for 1–2 hrs at 4°C. In GluN2B binding assays, GST was used to assess total protein and bound CaMKII levels were determined by the ratio of bound to total CaMKII. Blots were developed using chemiluminescence (Super Signal West Femto,Thermo-Fisher) and imaged using the Chemi-Imager 4400 system (Alpha-Innotech). Densitometry was calculated in AlphaEaseFC (Alpha-Innotech) (Barcomb et al., 2014; Goodell et al., 2014, 2016). The relative immuno-detection value (IDV) was normalized as a percent of the average of WT conditions for the same blot, which was set at a value of one to allow comparison between multiple experiments. Sample size is indicated by individual data points on graph and statistical analysis is detailed in figure legends.

#### Fluorescence correlation spectroscopy

Fluorescence correlation spectroscopy (FCS) analysis was performed on a Zeiss LSM780 spectral microscope equipped with ZEN2011 software and an FCS/RICS package. 50nM fluorescein was used to optimize the microscope pinhole position and to determine the structural parameter of the detection volume, calculated to be 7.4. Extracts from HEK293 cells were diluted to achieve a baseline count rate of 5,000–50,000 counts per second (CPS; kHz) to achieve a particle number (N) of ≤10 (autocorrelation curve y-intercept ≥ 0.1 when curve decays to 0). Ten acquisitions were performed for 10 seconds each and averaged together for a single n. Traces with obvious indications of protein aggregates were excluded from molecular brightness analysis. Aggregates were defined as spikes in the fluorescence intensity trace that reached over two times the average deviation in fluorescence intensity. Autocorrelation curves were fit with a 3D Gaussian distribution model using PyCorrFit software. Molecular brightness is reported as counts per particle (kHz). Experiments and analyses were performed with the observer blinded to the mutation being analyzed. Sample size is indicated by individual data points on graph and statistical analysis is detailed in figure legends.

### Quantification and statistical analysis

Data are shown as mean ± SEM. All data were tested for ability to meet parametric assumptions including normality, homoscedasticity, and independence. Statistical significance and sample size (n) are indicated in the figure legends. Data obtained from imaging experiments were obtained using SlideBook 6.0 software (3i) and analyzed using Prism (GraphPad) software. All data met parametric conditions, as evaluated by a Shapiro-Wilk test for normal distribution and a Brown-Forsythe test (3 or more groups) or an F-test (2 groups) to determine equal variance. Comparisons between two groups were analyzed using unpaired, two-way Student's t tests. Comparisons between pre- and post-treatment images from the same cells were analyzed using paired, two-way Student's t tests. Comparisons between three or more groups were done by one-way ANOVA with Tukey's post hoc analysis, unless indicated otherwise. Statistical significance is indicated, including by ∗p<0.05; ∗∗p<0.01; ∗∗∗p<0.001, ∗∗∗∗p<0.0001, or n.s., not significant.

## Data Availability

Data are available at Mendeley Data, V1, https://doi.org/10.17632/t5zhhhrfb9.1.
