# Supplementary material for: Characterization of six CaMKIIα variants found in patients with schizophrenia
Source: iScience. 2021 Sep 27;24(10):103184. doi: 10.1016/j.isci.2021.103184 (PMC8506966; doi:10.1016/j.isci.2021.103184)
Supplement: Document S1. Figure S1 and Table S1 [file mmc1.pdf]

iScience, Volume *24*

Supplemental information

Characterization of six CaMKIIα variants

found in patients with schizophrenia

Carolyn Nicole Brown, Sarah G. Cook, Hillary F. Allen, Kevin C. Crosby, Tarjinder Singh, Steven J. Coultrap, and K. Ulrich Bayer

Table S1: Information on the CaMKII variants studied, related to Figure 1. The databases SCHEMA (schizophrenia), BiPex (bipolar disorder), and Epi25 (epilepsy) can be found on the webpage of the Broad Institute. The annotations of CaMKII variants in the database can differ, as they are based on the minor splice variant αB, which contains a 33 nt / 11 aminoacids insert. In the overall variant counts, only variants that change amino acids are counted; the main number indicates the number of distinct variants, the number in brackets indicates the total counts of variants detected.

					counts in SCHEMA		***counts in BiPex***		***counts in Epi25***	
**variant**	**name in database**	**position GRCh37**	original codon	variant codon	**patients (of 48496)**	**controls (of 194644)**	**patients (of 27866)**	**controls (of 28844)**	**patients (of 18340)**	**controls (of 16820)**
R8H	pArg8His	5-149669166-C-T	CGA	CAC	1	0				
P242L	pPro242Leu	5-149630342-G-A	CCG	CUG	1	2			0	1
R396st	pArg407ter	5-149607773-G-A			1	0				
R433C	pArg444Cys	5-149602688-G-A	CGC	UGC	1	0				
R457C	pArg468Cys	5-149602616-G-A	CGC	UGC	1	1				
R477H	pPro488His	5-149602555-G-T	CCC	CAC	1	0				
**number of variants only in patients or controls**					18 (18)	36 (41)	8 (8)	5 (6)	9 (9)	10 (11)
**number of variants in both**					10 (11)	10 (18)	2 (6)	2 (5)	0	0
**total number of variant occurances**					29	59	14	11	9	11
**fold frequency in patients over controls**					1.97		1.32		0.75	

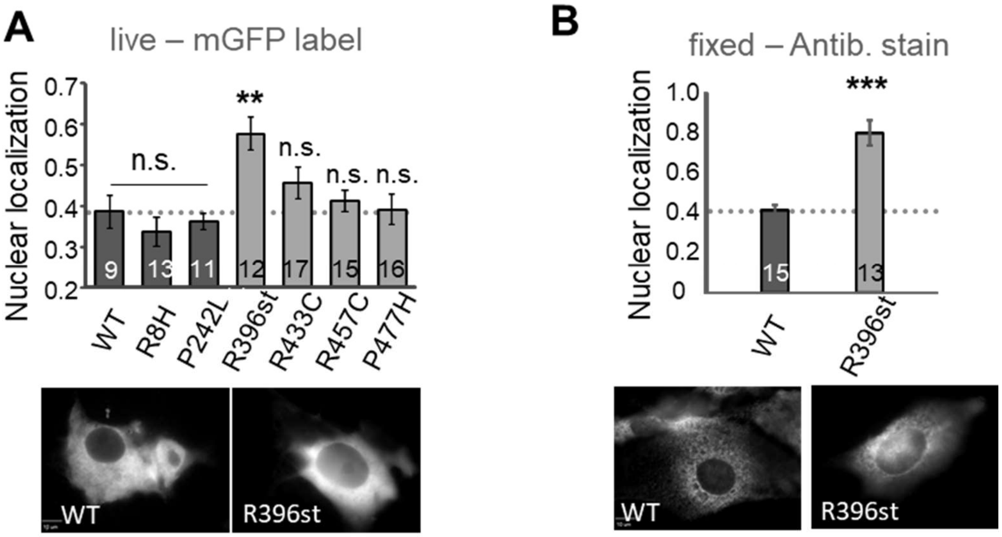

Figure S1: The R396st variant has increased nuclear localization comparted to WT, related to Figures 2 and 3. CaMKII variants were expressed in HEK293 cells. Data are represented as mean +/- SEM.

(A) GFP-CaMKII wild type (WT) and variants were imaged in live cells, and their ratio of nuclear over cytoplasmic localization was quantified. The ratio for the R396st variant is still below one (indicating preferential cytoplasmic localization), but significantly increased compared to WT and all other variants tested; **$p<0.01$ in one-way ANOVA with Tukey's post hoc analysis.

(B) Unlabelled CaMKII WT or R396st variant was detected by immunostaining in fixed cells. Again, the R396st was still preferentially cytoplasmic (with a nuclear to cytoplasm localization ratio below one) but with significantly more nuclear localization compared to WT; ***$p<0.001$ in two-tailed Student's t-test.
